# Compartmentalized Cyclic AMP Production by the *Bordetella pertussis* and *Bacillus anthracis* Adenylate Cyclase Toxins Differentially Affects the Immune Synapse in T Lymphocytes

**DOI:** 10.3389/fimmu.2018.00919

**Published:** 2018-05-01

**Authors:** Vijay B. Arumugham, Cristina Ulivieri, Anna Onnis, Francesca Finetti, Fiorella Tonello, Daniel Ladant, Cosima T. Baldari

**Affiliations:** ^1^Department of Life Sciences, University of Siena, Siena, Italy; ^2^Neuroscience Institute, Italian National Research Council, Padua, Italy; ^3^Department of Structural Biology and Chemistry, Institut Pasteur, Paris, France

**Keywords:** immune synapse, cyclic AMP, compartmentalization, T-cell antigen receptor signaling, adenylate cyclase toxin

## Abstract

A central feature of the immune synapse (IS) is the tight compartmentalization of membrane receptors and signaling mediators that is functional for its ability to coordinate T cell activation. Second messengers centrally implicated in this process, such as Ca^2+^ and diacyl glycerol, also undergo compartmentalization at the IS. Current evidence suggests a more complex scenario for cyclic AMP (cAMP), which acts both as positive and as negative regulator of T-cell antigen receptor (TCR) signaling and which, as such, must be subjected to a tight spatiotemporal control to allow for signaling at the IS. Here, we have used two bacterial adenylate cyclase toxins that produce cAMP at different subcellular localizations as the result of their distinct routes of cell invasion, namely *Bordetella pertussis* CyaA and *Bacillus anthracis* edema toxin (ET), to address the ability of the T cell to confine cAMP to the site of production and to address the impact of compartmentalized cAMP production on IS assembly and function. We show that CyaA, which produces cAMP close to the synaptic membrane, affects IS stability by modulating not only the distribution of LFA-1 and its partners talin and L-plastin, as previously partly reported but also by promoting the sustained synaptic accumulation of the A-kinase adaptor protein ezrin and protein kinase A while suppressing the β-arrestin-mediated recruitment of phosphodiesterase 4B. These effects are dependent on the catalytic activity of the toxin and can be reproduced by treatment with a non-hydrolyzable cAMP analog. Remarkably, none of these effects are elicited by ET, which produces cAMP at a perinuclear localization, despite its ability to suppress TCR signaling and T cell activation through its cAMP-elevating activity. These results show that the IS responds solely to local elevations of cAMP and provide evidence that potent compartmentalization mechanisms are operational in T cells to contain cAMP close to the site of production, even when produced at supraphysiological levels.

## Introduction

High concentrations of cyclic AMP (cAMP) are known to suppress the activation and function of immune cells (1). Several bacterial pathogens exploit this property to evade the immune response by producing cAMP-elevating toxins, as exemplified by the ADP-ribosylating toxins of *Vibrio cholerae* or *Bordetella pertussis* (PT), which promote the accumulation of cAMP by activating cellular Gs proteins or inactivating cellular Gi proteins, respectively (2, 3), or the adenylate cyclase (AC) toxins produced by *Bordetella pertussis* (CyaA), *Bacillus anthracis* [edema toxin (ET)], or *Pseudomonas aeruginosa* (ExoY), which directly catalyze cAMP production in infected cells (4–6).

T lymphocytes are among the cellular targets of bacterial AC toxins (7, 8). We have previously reported that both CyaA and ET suppress T-cell antigen receptor (TCR) signaling and T cell activation and proliferation through their cAMP-elevating activity (9, 10) and impair T cell migration by inhibiting chemokine receptor signaling (9, 11). At low concentrations, they moreover instruct CD4 T cells to differentiate to Th2 and Th17 effectors by selectively affecting the activation of specific components of the TCR signaling cascade (12, 13). Of note, we have shown that CyaA binds to LFA-1, which allows for its accumulation at the highly specialized signaling platform that forms at the interface of T cells with cognate antigen-presenting cells (APCs), known as the immune synapse (IS) (14, 15). Following its internalization, CyaA is retained at a subsynaptic localization, catalyzing the production of cAMP which suppresses TCR signaling and promotes the protein kinase A (PKA)-dependent disengagement of LFA-1 from the IS, which results in IS disassembly (14).

Compartmentalization of membrane receptors and intracellular signaling molecules in the concentric synaptic subdomains of the central, peripheral, and distal supramolecular activation clusters (SMAC) is one of the most striking features of the IS, on which its function in the orchestration of the signals that drive T cell activation crucially depends (15). Remarkably, compartmentalization extends to second messengers that are generated at the IS, including Ca^2+^, diacyl glycerol, and PIP3 (16–19). This is achieved through the tight spatiotemporal control of the enzymes responsible for their generation as well as for their degradation, as exemplified for cAMP (20). TCR engagement results in the transient accumulation of cAMP (21), which is essential at the initial steps of the signaling cascade to promote the EPAC1-dependent activation of Rap1 (22), a small GTPase implicated in inside-out signaling by the TCR to induce the open, active conformation of LFA-1 that is essential to stabilize the IS (23, 24). TCR engagement also promotes the synaptic accumulation of ezrin (25, 26), an actin-binding protein and a member of the A-kinase adaptor proteins (AKAP) that recruits PKA to the IS, where it can be locally activated by cAMP (27). PKA has the ability to negatively regulate TCR signaling by enhancing the activity of the kinase Csk, which inhibits the initiating kinase Lck (28). However, in the presence of costimulation by CD28, which accumulates at the cSMAC together with the TCR (15), premature termination of TCR signaling is prevented by the local degradation of cAMP through the β-arrestin-dependent recruitment of phosphodiesterase 4B (PDE4B) (29). Moreover PKA is displaced from the IS to the opposite pole of the cell (30, 31), allowing signaling to proceed for the sustained timeframe required for T cell activation.

Although these findings support the notion that cAMP is produced and confined close to engaged TCRs to locally modulate signaling, a formal proof that T cells are indeed able to limit cAMP diffusion from the sites of production within their scant cytoplasm such that only the molecules within specific cAMP domains can respond is as yet lacking. Here, we have compared the ability of T cells to assemble a functional IS, used as a readout of a process where signaling is orchestrated within the restricted area of the synaptic membrane, following intoxication with *B. pertussis* CyaA or *B. anthracis* ET. While both these toxins catalyze a robust elevation in cAMP, they exploit different mechanisms to enter the host cell, leading to the generation of cAMP domains at different subcellular localizations. Namely, after binding to its cell target CyaA remains associated with the plasma membrane, catalyzing the generation of a plasma membrane-proximal cAMP pool. At variance, after its receptor-mediated internalization at the plasma membrane ET catalyzes the production of cAMP at a perinuclear localization, where the toxin is released from late endosomes, remaining associated with their cytosolic face (32, 33). We show that CyaA impairs IS stability not only by inducing the disengagement of LFA-1 and its partners talin and L-plastin but also by promoting the sustained synaptic accumulation of ezrin and PKA while suppressing the recruitment of β-arrestin and PDE4B. These effects are dependent on the cAMP-elevating activity of the toxin and can be reproduced by a non-hydrolyzable cAMP analog. Remarkably, none of these effects are elicited by ET, despite its ability to suppress TCR signaling and T cell activation in a cAMP-dependent fashion, indicating that the IS can only be modulated by locally generated cAMP domains and supporting the notion that T cells are able to contain cAMP close to the site of production, even within the limited range of their scant cytoplasm.

## Materials and Methods

### Cells, Antibodies, and Reagents

Primary T cells were purified from buffy coats from anonymous healthy donors using the StemSep Human T cell enrichment kit (Voden Medical InstrumentsSpA). Raji B-cells (of human origin) were used as APC.

Anti-talin (clone 8d4), anti-ezrin (E1281), and anti-PDE4B (sc-25812) antibodies were purchased from Sigma-Aldrich. Anti-PDE4B (sc-25812), anti-P-Erk (T202/Y204), and anti-Erk2 antibodies were purchased from Santa Cruz Biotechnology. Anti-CD11a (27/CD11a) mAb and PE-labeled anti-CD3, FITC-labeled anti-CD69 mAb, and PE-labeled anti-CD25 mAb were purchased from BD Biosciences. Anti-β-arrestin-2 (ab54790) mAb was from Abcam. Anti-L-Plastin (clone LPL4A.1), PE-labeled phalloidin (A34055) and Dynabeads^®^ Human T-Activator CD3/CD28 were from Thermo-scientific. AlexaFluor 488- and Alexa Fluor 555-labeled secondary antibodies were from Molecular Probes. Anti-phosphotyrosine mAb (clone 4G10) was from Millipore.

SEB and SEE were purchased from Toxin Technology and handled in accordance with the specific hazard and safe handling practices. Cell Tracker Blue was purchased from Molecular Probes. Poly-l-lysine, 8-CPT-cAMP, and PP2 were purchased from Sigma-Aldrich. Carboxyfluorescein succinimidyl ester (CFSE) was purchased from Molecular Probes Europe BV (Leiden, The Netherlands).

### Toxins

CyaA and its enzymatically inactive variant CyaA-E5 (resulting from a Leu-Gln dipeptide insertion between D188 and I189 in the catalytic core of the enzyme) were expressed in *E. coli* and purified to near homogeneity by previously established procedures modified as described in order to eliminate most of the contaminating endotoxin (<0.5 EU/μg protein) (34). The specific activity of CyaA, measured as described in Ladant et al. (35) was higher than 500 µmol cAMP/min/mg whereas CyaA-E5 had no detectable enzymatic activity. PA, EF, and EF-K346R (mEF), an AC mutant form of EF with 10,000-fold less catalytic activity (36) were expressed in *E. coli* as N-terminal His-tag fusions in BL21 DE3 (Novagen Inc.) and purified by Ni-charged Hitrap chelating (Amersham Biosciences) as described (32, 37). Adenylyl cyclase activity was measured in the presence of 10 mM ATP, 10 µM CaM, 1.2 µM free CaCl_2_, 500 µM BAPTA, and 10 mM MgCl_2_ as described (38). Toxins were handled in accordance with biosafety handling practices.

### Cell Intoxication and cAMP Measurements

For cAMP and proliferation assays, T cells were intoxicated with 45 nM CyaA or mCyaA by incubation in serum-free RPMI 1640 (supplemented with 2 mM CaCl_2_) at 37°C for 15–30 min. For intoxication with ET or mET, the binding-competent toxin was first assembled by incubation of the receptor-binding subunit protective antigen PA (19 nM) with the cataytic subunit EF or its inactive mutant (11 nM) (ratio PA:EF or mEF = 1.6) for 15–30 min at 37°C. T cells were then intoxicated with ET or mET by incubation in complete medium at 37°C for 2–4 h. Under these conditions maximal cAMP levels were reached, as previously reported (9, 10, 12). For conjugate experiments, T cells were incubated with 45 nM CyaA or mCyaA in serum-free RPMI 1640 (supplemented with 2 mM CaCl_2_) on ice for 15 min to allow binding, then washed, resuspended in phosphate-buffered saline (PBS), mixed with APCs (1:1), and shifted to 37°C as described (14). Alternatively, T cells were intoxicated with ET/mET for 4 h as described above, then washed, resuspended in PBS, mixed with APCs (1:1), and shifted to 37°C.

Intracellular cAMP was quantified by enzyme-linked immunoassay (Biotrak EIA; Amersham Biosciences) according to the manufacturers’ instructions. For these experiments, 2 × 10^6^ cells T cells were plated in 96-well plates in 200 µl RPMI 1640-7.5% FCS and treated with CyaA/mCyaA or ET/mET as described above. At the end of the treatment, cells were washed 2× in PBS and lysed in the lysis reagent included in the kit.

### Conjugate Formation and Confocal Microscopy

Conjugate formation was carried out as described (39). Briefly, Raji cells (used as APC) were pulsed for 2 h with 10 µg/ml SEE/SEB and labeled with 10 µM Cell Tracker Blue for the last 20 min. They were then washed, mixed with T cells (1:1), either untreated or intoxicated as described above, incubated at 37°C for 15 min and plated on polylysine-coated wells of diagnostic microscope slides (Erie Scientific Company). Alternatively, 100 µM of 8-CPT was added 2 min after the start of conjugate formation. Cells were allowed to adhere for 15 min and then fixed in methanol at −20°C for 10 min or in 4% paraformaldehyde for 20 min at RT. Antigen-independent conjugates were obtained by mixing T cells and APC in the absence of SEE/SEB. Representative images of the latter after staining with the mAbs used are shown in Figure S1 in Supplementary Material. Where required due to a strong antibody signal at the APC side of the IS, 8 × 10^4^ T cells were resuspended in 30 µl medium, added with 2 µl of washed Dynabeads^®^ Human T-Activator CD3/CD28 to obtain a bead-to-cell ratio of 1:1 according to the manufacturer’s instructions, incubated at 37°C for 15 min and plated on polylysine-coated wells as above.

After fixation, samples were processed for immunofluorescence as previously described (39). Confocal microscopy was performed on a LSM700 (Carl Zeiss, Inc.) using a 63× objective. Z series of optical sections were performed at 0.5-µm increments. Images to quantify were acquired with pinholes opened to obtain 0.8-μm-thick sections. Detectors were set to detect an optimal signal below the saturation limits. Images were processed with Zen 2009 image software (Carl Zeiss, Inc.).

Scoring of conjugates for the polarization of the molecule of interest the IS was based on the presence of staining selectively concentrated at the T-cell contact with the APC compared to the remaining T cell area. Clustering of molecules at the T-cell/APC contact site was quantified on median optical confocal sections using ImageJ software (National Institutes of Health). Namely, fluorescence intensity of the molecule at the IS area was divided by the intensity calculated for the entire cell and is expressed as relative recruitment index as previously described (39). The quantitative colocalization analysis was performed on median optical sections using ImageJ and JACoP plug-in to determine Manders’ coefficient.

### T Cell Activation and Proliferation Assays, Immunoblots

Proliferation was measured by flow cytometry using the CFSE dilution method. Briefly, T cells were resuspended at 20 × 10^6^/ml in PBS and stained with 10 µM CFSE for 8 min at RT. Cells were subsequently washed twice in RPMI-7.5% fetal calf serum, resuspended at a concentration of 5 × 10^6^/ml and either left untreated or intoxicated with CyaA, ET, or the respective mutants, or treated with 8-CPT, as described above. Raji cells were pulsed for 2 h with 10 µg/ml SEE/SEB, fixed with 0.05% glutaraldehyde PBS for 30 s, added with 0.2 M glycine in PBS to block the reaction, washed twice with PBS-1% BCS and resuspended in RPMI-7.5% FCS at a concentration of 5 × 10^6^/ml. T cells and Raji cells were then mixed in 12-well plates at a ratio of 1:1, incubated at 37°C for 24–120 and analyzed by flow cytometry after gating on lymphocytes and counterstaining with PE-labeled anti-CD3 mAb. T cell activation was assessed by flow cytometric analysis of the activation markers CD69 and CD25 on cells either left untreated or intoxicated with CyaA, ET or the respective mutants, or treated with 8-CPT, and activated with SAg-pulsed fixed Raji cells for 16–24 h. For the immunoblot analysis of Erk activation T cells were intoxicated and activated as detailed above for 15 min. Post-nuclear supernatants were processed for immunoblot as described (11).

### Statistical Analyses

Mean values, SD values, and Student’s *t* test (unpaired) were calculated using the Microsoft Excel application. One-way ANOVA followed by Tukey *post hoc* test was used for multiple comparisons and calculated using GraphPad (Prism Software). A *P* value <0.05 was considered as statistically significant.

## Results

### LFA-1 Accumulation at the IS Is Compromised in T Cells Intoxicated With CyaA, but Not With ET

**Figure 1:**
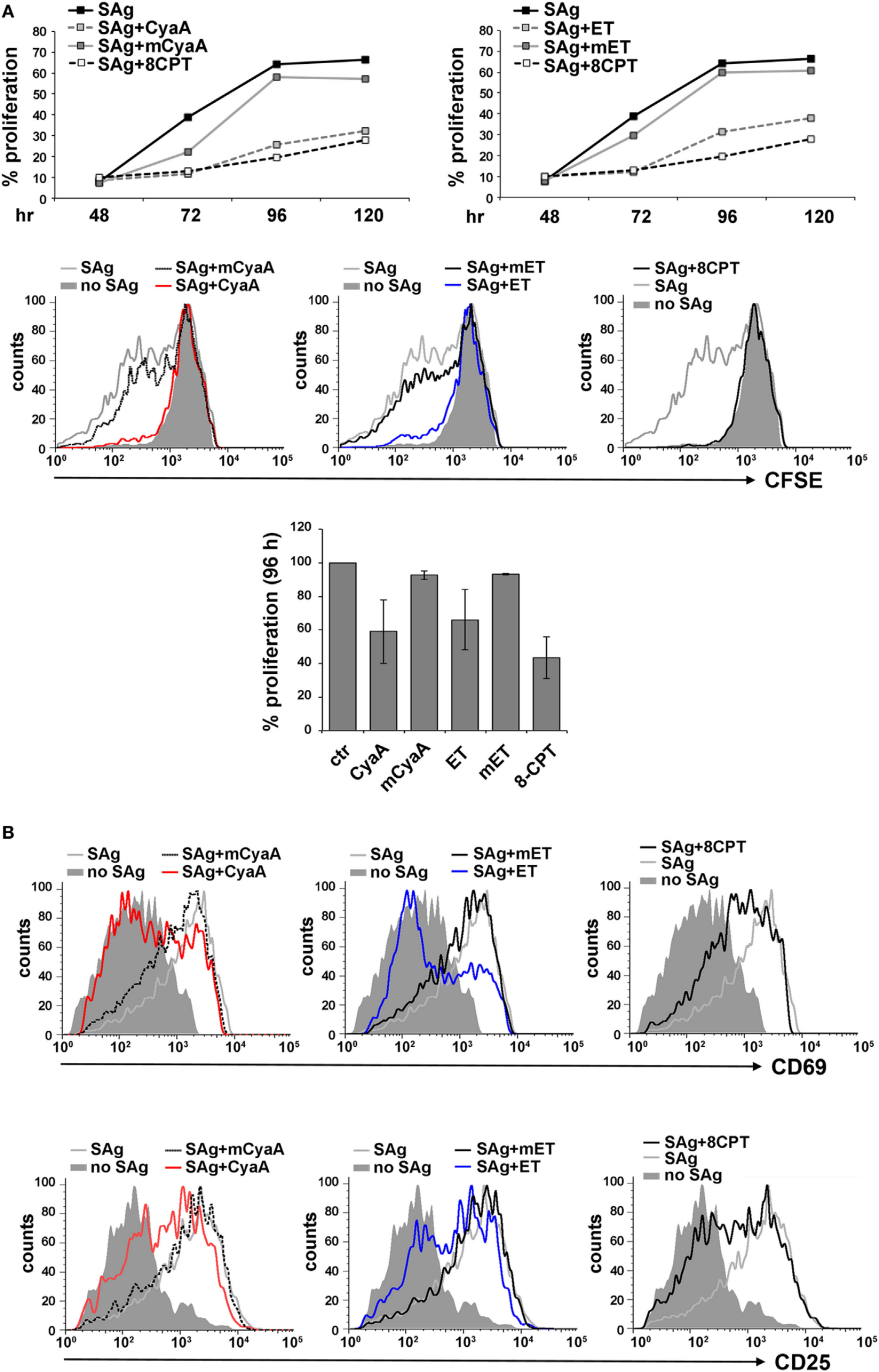

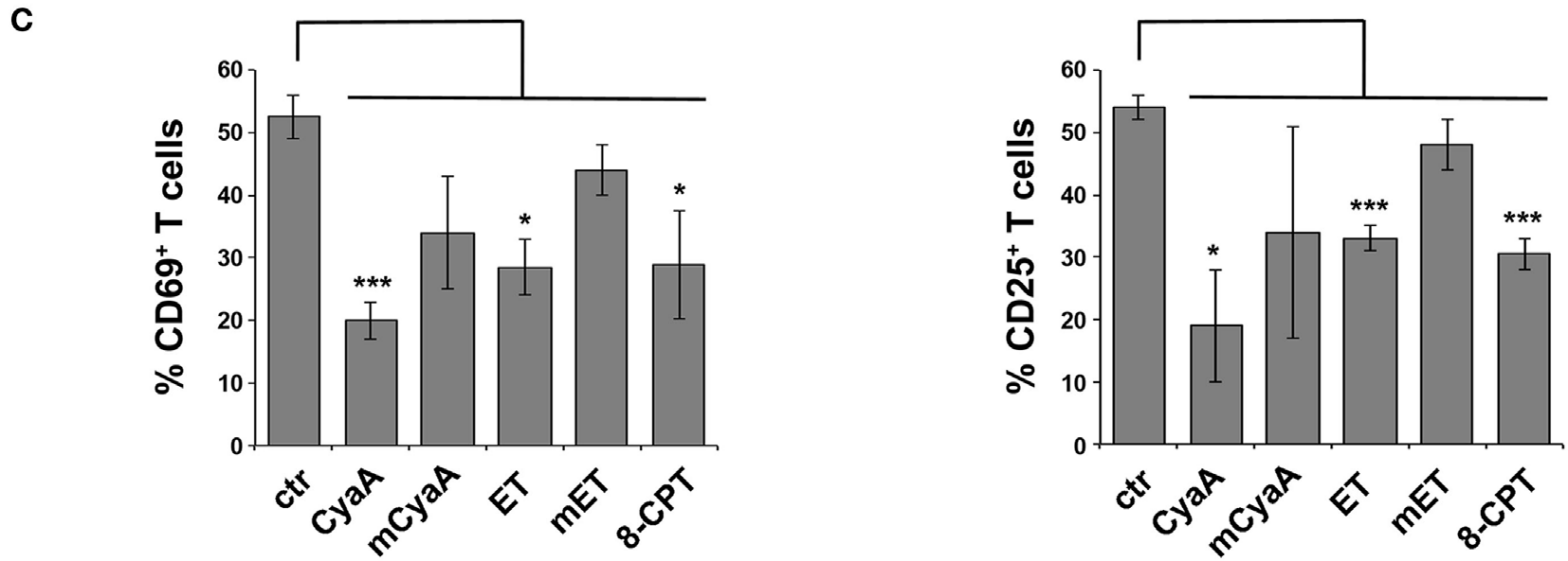
CyaA and edema toxin (ET) suppress T cell activation and proliferation. **(A)** Top, flow cytometric analysis of carboxyfluorescein succinimidyl ester (CFSE)-labeled peripheral blood mononuclear cells stimulated for 48, 72, 96, or 120 h with glutaraldehyde-fixed SAg-pulsed antigen-presenting cell (APC). Cells were incubated for 15 min on ice with 45 nM of either CyaA or mCyaA, or for 4 h at 37°C with ET (19 nM PA + 11 nM EF) or mET (19 nM PA + 11 nM mEF) before washing and mixing with the APCs. Alternatively, 100 µM 8-CPT was added 2 min after cells were mixed with APCs. Cells were labeled with anti-CD3 mAb to identify T cells. The histograms show the percentage of T cells having undergone proliferation (CD3^+^CFSE^low^) in one of the two donors analyzed. Middle, FACS profiles of the same samples at the 96 h time point. Bottom, histograms showing the percentage of T cells having undergone proliferation (CD3^+^CFSE^low^) at the 96 h time point in the two donors analyzed (mean ± SD). **(B,C)** Flow cytometric analysis of purified peripheral blood T cells stimulated for 16 h (CD69) or 24 h (CD25) with glutaraldehyde-fixed SAg-pulsed APC in the presence or absence of 45 nM CyaA/mCyaA or 11 nM ET/mET or 100 µM 8-CPT as described in **(A)**. Cells were labeled with either anti-CD69 (top) or anti CD25 (bottom) mAbs. FACS profiles of a representative donor are shown in **(B)**. The histograms **(C)** show the percentage of CD69^+^ (left) or CD25^+^ (right) T cells (mean ± SD, three donors; Student’s *t* test).

To address the susceptibility of the IS to cAMP pools generated at different subcellular localizations, peripheral T cells purified from healthy donors were first intoxicated with CyaA or ET. Since we had previously shown that the kinetics of cAMP production by CyaA in T cells is faster compared to ET (9, 12), cells were intoxicated with CyaA or ET for 30 min and 2 h, respectively, to reach the peak of cAMP production. Under these conditions, high levels of cAMP were measurable in both CyaA- and ET-treated T cells (Figure S2A in Supplementary Material). Consistent with our previous report, T cell proliferation was suppressed by both toxins, as assessed by flow cytometric analysis of CyaA or ET-intoxicated T cells loaded with CFSE and mixed with Raji cells, used as APC, pulsed with a mix of staphylococcal enterotoxins B and E (SEB + SEE; SAg) which together activate a major proportion of the human TCR Vβ repertoire (Figure 1A). In addition, T cell activation was inhibited by both toxins, as assessed by flow cytometric analysis of the activation markers CD69 and CD25 (Figures 1B,C), indicating that the cAMP produced by the toxins interferes with T cell activation upstream of the autocrine IL-2/IL-2 receptor loop on which their proliferation depends. Moreover, immunoblot analysis showed that both toxins were able to inhibit the activation of Erk, a downstream component of the TCR signaling cascade (Figure S2B in Supplementary Material).

**Figure 2:**
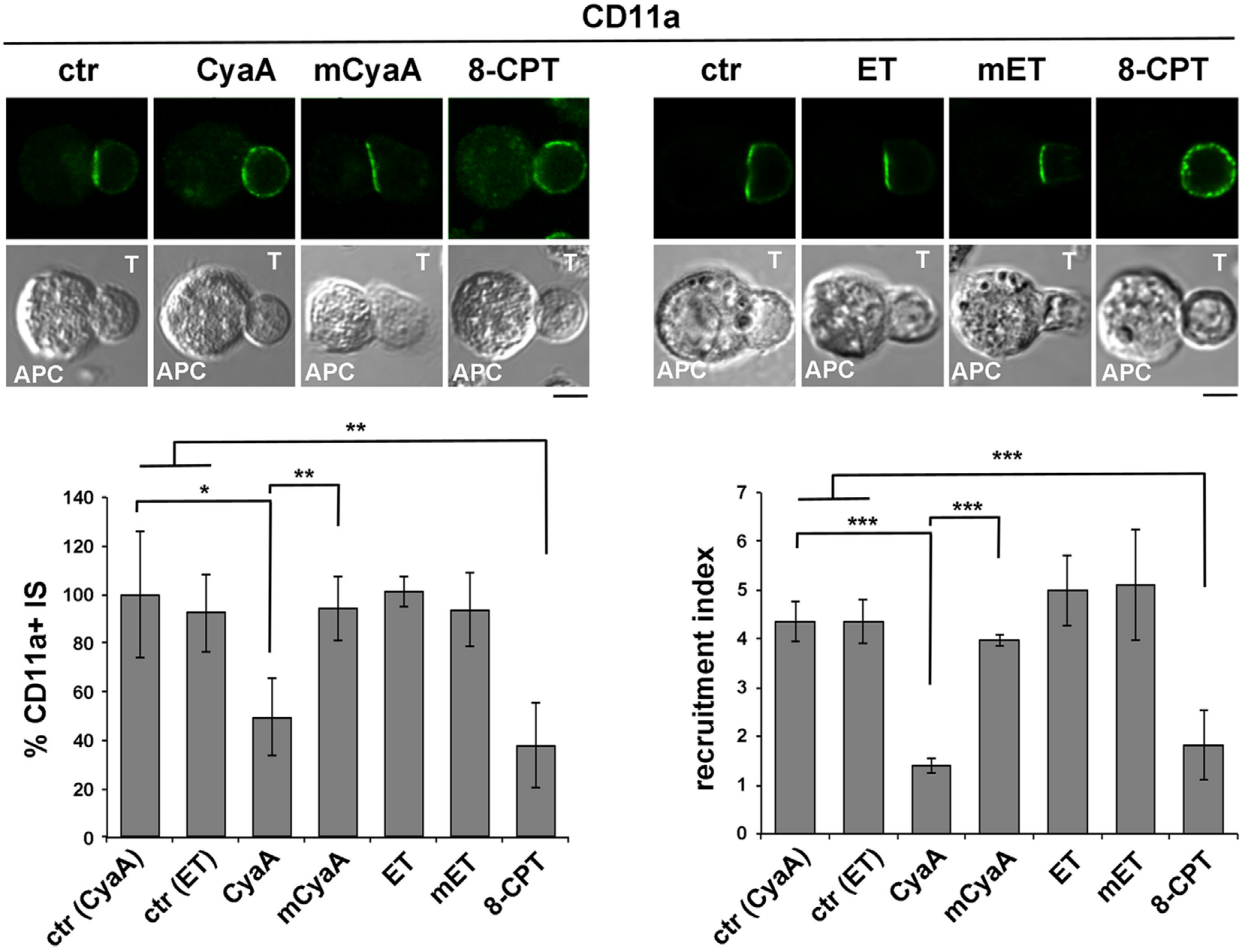
LFA-1 accumulation at the immune synapse (IS) is compromised in T cells intoxicated with CyaA, but not with edema toxin (ET). Immunofluorescence analysis of LFA-1 (anti-CD11a mAb) in conjugates of peripheral T cells and SAg-pulsed antigen-presenting cell (APC), incubated at 37°C for 15 min and allowed to adhere on polylysine-coated wells for further 15 min. For intoxication, T cells were incubated for 15 min on ice with 45 nM of either CyaA or mCyaA, or for 4 h at 37°C with ET (19 nM PA + 11 nM EF) or mET (19 nM PA + 11 nM mEF) before washing and mixing with the APC. Ctrl cells were left untreated. Alternatively, 100 µM 8-CPT was added 2 min after T cells were mixed with APCs. Histograms show the percentage of conjugates harboring CD11a at the IS (mean ± SD, *n* = 3 donors; left) and the relative CD11a fluorescence at the T-cell:APC contact site compared with the remaining T cell area (expressed as relative recruitment index; right) (*n* = 45 cells, 15 conjugates/donor). Due to the different efficiency of conjugate formation among donors, the percentages of synapses positive for CD11a in treated vs untreated cells in each experiment have been normalized taking the value of untreated controls as 100%. The range of CD11a^+^ synapses in untreated controls from the three donors is ~50–90%. Representative images are shown. Size bar, 5 µm. *P < 0.05; **P < 0.01; ***P < 0.005; ****P < 0.001. Error bars, SD (one-way ANOVA).

To compare the impact of cAMP production by CyaA and ET on LFA-1 accumulation at the IS, conjugates of CyaA- or ET-intoxicated T cells and SAg-loaded APCs were imaged by confocal microscopy following staining with anti-LFA-1 mAb. As previously reported, under these conditions LFA-1 was found to be distributed evenly along the plasma membrane in CyaA-treated T cells, at variance with the tight synaptic accumulation in non-intoxicated T cells (Figure 2). This effect, which we had previously shown to result from disengagement from the IS of LFA-1 that had initially accumulated at this location (14), was dependent on the cAMP-elevating activity of CyaA, as it was not reproduced by the catalytically defective CyaA-E5 mutant (mCyaA) (Figure 2). Strikingly, ET did not affect the synaptic accumulation of LFA-1 (Figure 2), despite the fact that cAMP accumulation in ET-intoxicated T cells was higher than in the same cells intoxicated with CyaA (Figure S2 in Supplementary Material). Interestingly, treatment of conjugates with the non-hydrolyzable cAMP analog, 8-CPT, which diffuses freely through the cell, reproduced the effects of CyaA (Figure 2), suggesting that signaling modules that control IS architecture are differentially affected by cAMP. Collectively, these results indicate that LFA-1 engagement at the IS is exquisitely susceptible to local cAMP and that diffusion to the IS of cAMP generated by ET at perinuclear late endosomes is prevented notwithstanding its high intracellular concentration.

### The Synaptic Accumulation of the LFA-1 Interactors, Talin, and L-Plastin, Is Compromised in T Cells Intoxicated With CyaA, but Not With ET

Signaling by TCRs that assemble into microclusters at the dSMAC promotes the polymerization of new actin filaments that assist the movement of engaged TCRs toward the center of the IS. While TCRs eventually concentrate at the cSMAC, F-actin accumulates at the pSMAC, stabilizing the T cell-APC conjugate by interacting with LFA-1 through the actin adaptor talin (40, 41). The Ca^2+^-calmodulin-dependent actin-bundling protein L-plastin is also recruited to the pSMAC, where it promotes the sustained accumulation of LFA-1 and talin, further contributing to IS stability (42, 43).

**Figure 3:**
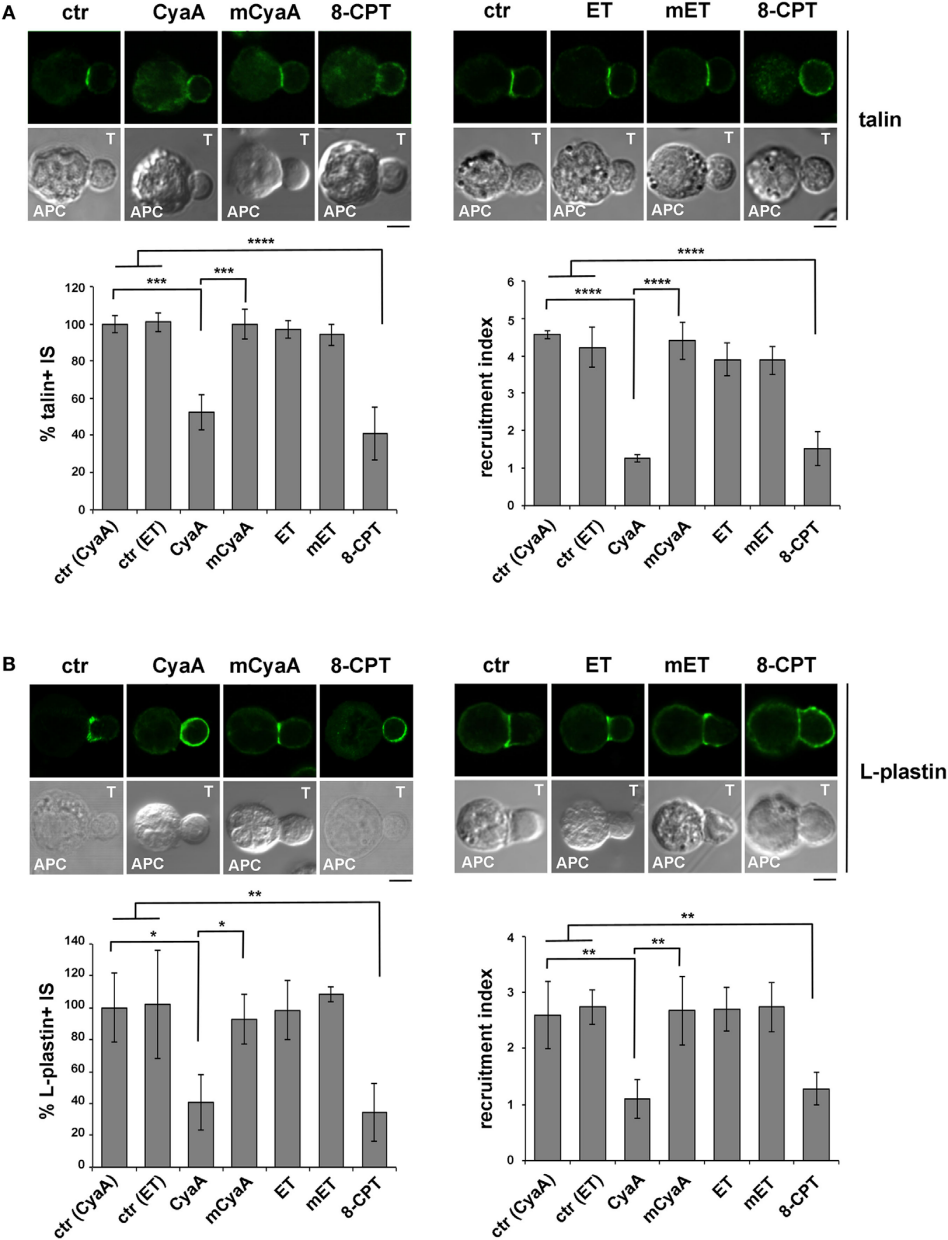

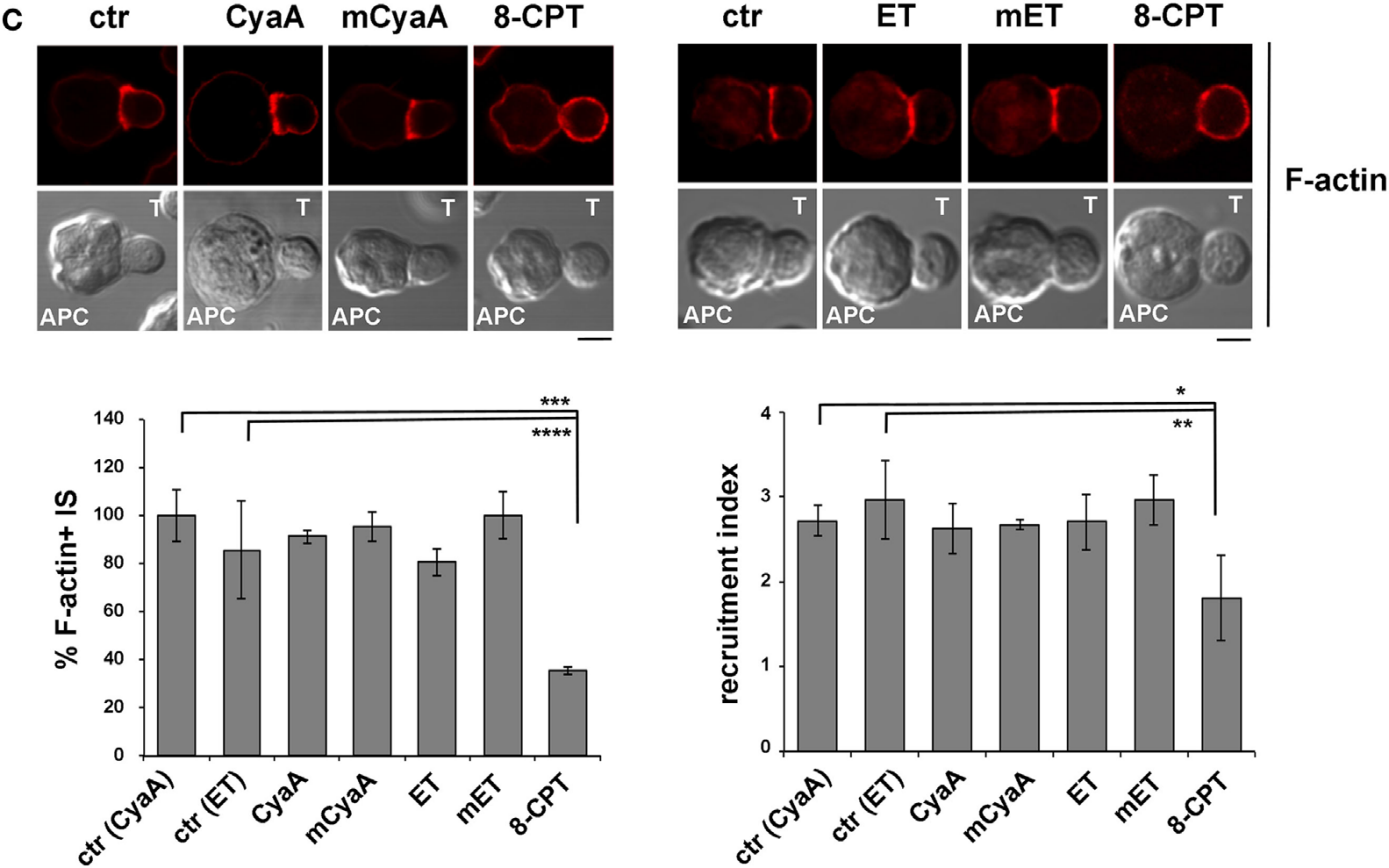
The synaptic accumulation of the LFA-1 interactors, talin, and L-plastin, is compromised in T cells intoxicated with CyaA, but not with edema toxin (ET). Immunofluorescence analysis of talin **(A)**, L-plastin **(B)**, or F-actin (phalloidin staining) **(C)** in conjugates of peripheral T cells and SAg-pulsed antigen-presenting cell (APC), incubated at 37°C for 15 min and allowed to adhere on polylysine-coated wells for further 15 min. For intoxication, T cells were incubated for 15 min on ice with 45 nM of either CyaA or mCyaA, or for 4 h with ET (19 nM PA + 11 nM EF) or mET (19 nM PA + 11 nM mEF) before washing and mixing with the APC. Ctrl cells were left untreated. Alternatively, 100 µM 8-CPT was added 2 min after T cells were mixed with APCs. Histograms show the percentage of conjugates harboring talin, L-plastin, or F-actin at the immune synapse (IS) (mean ± SD, *n* = 3 donors; left), and the relative talin, L-plastin, or F-actin fluorescence at the T-cell:APC contact site compared with the remaining T cell area (expressed as relative recruitment index; right) (*n* = 45 cells, 15 conjugates/donor). Due to the different efficiency of conjugate formation among donors, the percentages of synapses positive for each marker in treated vs untreated cells in each experiment have been normalized taking the value of untreated controls as 100%. The range of talin-, L-plastin-, and F-actin-positive synapses in untreated controls from the three donors is ~70–80%, 40–60%, and 50–70%, respectively. Representative images are shown. Size bar, 5 µm. *P < 0.05; **P < 0.01; ***P < 0.005; ****P < 0.001. Error bars, SD (one-way ANOVA).

Consistent with our previous report (14), the synaptic accumulation of talin was found to be impaired in T cells intoxicated with CyaA (Figure 3A). This effect was reproduced by 8-CPT but not by mCyaA (Figure 3A). At variance, talin was found to accumulate normally at the IS formed by ET-intoxicated T cells (Figure 3A). Similar results were obtained when the distribution of L-plastin was analyzed in SAg-specific conjugates of CyaA or ET-intoxicated T cells (Figure 3B). Together, these findings provide evidence that cAMP compromises the synaptic accumulation of LFA-1 and its interactors talin and L-plastin only when produced from a plasma membrane-proximal localization. Of note, while F-actin accumulation at the IS was affected by neither CyaA, as previously reported (14), nor EF, it was impaired in the presence of 8-CPT (Figure 3C; Figure S4A in Supplementary Material). The fact that a robust accumulation of F-actin at the APC was observed in SAg-specific conjugates prior to 8-CPT addition (Figure S3A in Supplementary Material) indicates that under these conditions 8-CPT promotes the dissipation of synaptic F-actin, suggesting that the diffuse distribution of this cAMP analog could result in the modulation of signaling mediators that control F-actin stability, as reported for other cell types (44, 45).

### Phosphotyrosine Signaling at the IS Is Impaired in T Cells Intoxicated With CyaA, but Not With ET

**Figure 4:**
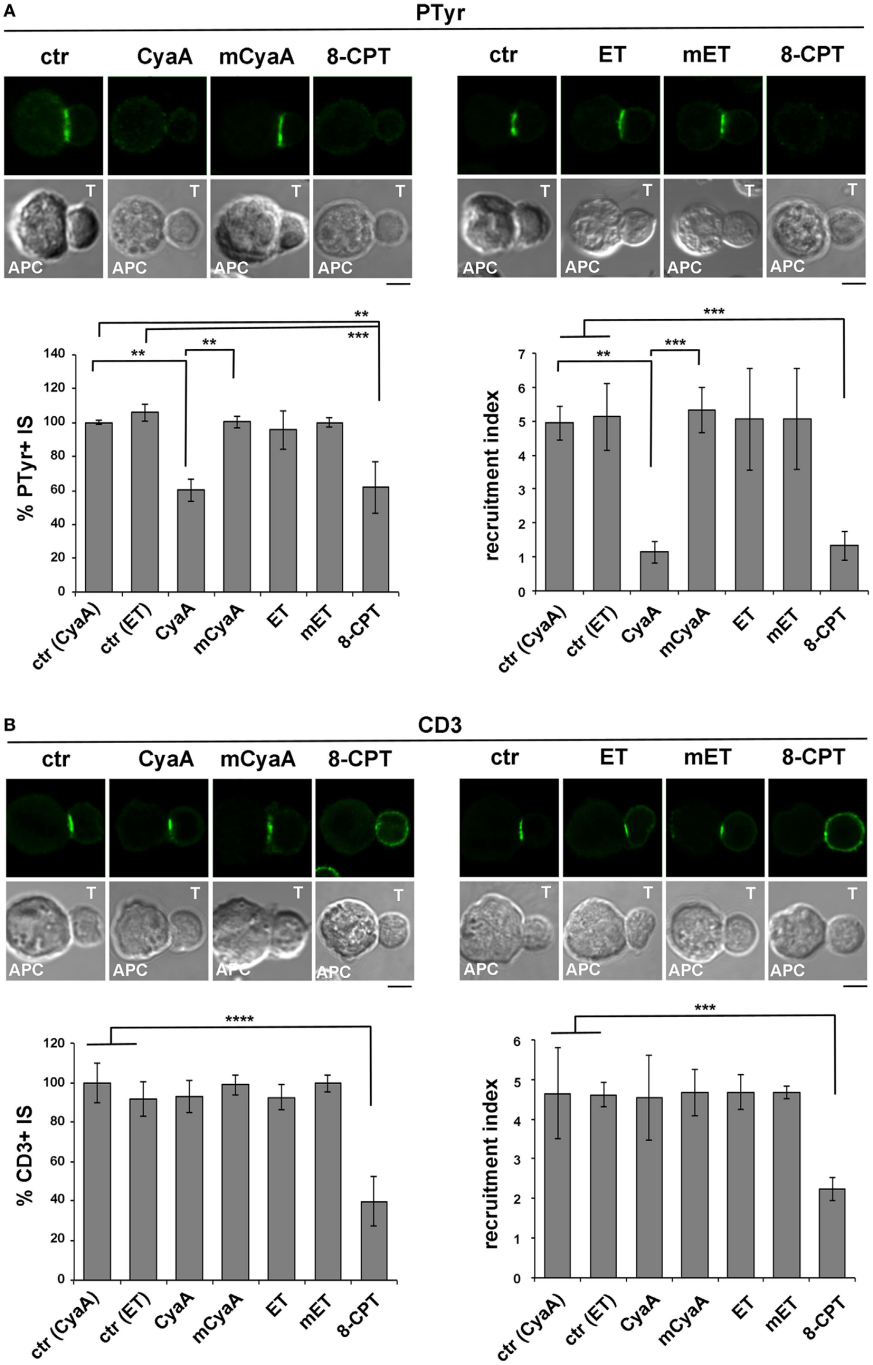
Phosphotyrosine signaling at the immune synapse (IS) is impaired in T cells intoxicated with CyaA, but not with edema toxin (ET). Immunofluorescence analysis of PTyr **(A)** or CD3ζ **(B)** in conjugates of peripheral T cells and SAg-pulsed antigen-presenting cell (APC), incubated at 37°C for 15 min and allowed to adhere on polylysine-coated wells for further 15 min. For intoxication, T cells were incubated for 15 min on ice with 45 nM of either CyaA or mCyaA, or for 4 h with ET (19 nM PA + 11 nM EF) or mET (19 nM PA + 11 nM mEF) before washing and mixing with the APC. Ctrl cells were left untreated. Alternatively, 100 µM 8-CPT was added 2 min after T cells were mixed with APCs. Histograms show the percentage of conjugates harboring PTyr or CD3ζ at the IS (mean ± SD, *n* = 3 donors; left) and the relative PTyr or CD3ζ fluorescence at the T-cell:APC contact site compared with the remaining T cell area (expressed as relative recruitment index; right) (*n* = 45 conjugates, 15 cells/donor). Due to the different efficiency of conjugate formation among donors, the percentages of synapses positive for each marker in treated vs untreated cells in each experiment have been normalized taking the value of untreated controls as 100%. The range of PTyr- and CD3ζ-positive synapses in untreated controls from the three donors is ~87–98 and 60–80%, respectively. Representative images are shown. Size bar, 5 µm. *P < 0.05; **P < 0.01; ***P < 0.005; ****P < 0.001. Error bars, SD (one-way ANOVA).

Immune synapse destabilization has been associated with a failure of T cells to undergo activation and proliferation as the result of impaired sustained TCR signaling (46). To understand whether the alterations in the accumulation of LFA-1 and its molecular partners at the IS resulted in defective TCR signaling, the synaptic accumulation of tyrosine phosphoproteins was imaged using an anti-phosphotyrosine antibody. A weak and diffuse PTyr signal was detected in CyaA-intoxicated or 8-CPT-treated T cell conjugates, as opposed to the strong and defined signal detected at the IS in SAg-specific conjugates of untreated T cells or T cells intoxicated with mCyaA, ET or mET (Figure 4A). Of note, the synaptic accumulation of the TCR was not affected by CyaA (Figure 4B), ruling out a defect of TCR recruitment to the IS as the cause of the impairment in phosphotyrosine signaling in CyaA-intoxicated T cells. At variance, 8-CPT treatment resulted in a failure of the TCR to remain associated with the IS despite its accumulation prior to 8-CPT addition (Figure S3B in Supplementary Material), as well as in premature termination of phosphotyrosine signaling (Figures S3C and S4B in Supplementary Material). These results suggest that by diffusing throughout the cell this cAMP analog may modulate mediators of TCR signaling and/or trafficking that are not in the normal range of the cAMP-enriched domain generated by CyaA at the IS.

### The Ezrin-Mediated Recruitment of PKA to the IS Is Enhanced in T Cells Intoxicated With CyaA

Having established that the IS is susceptible to CyaA but not to ET, we next investigated the impact of the IS-proximal production of cAMP by CyaA on the recruitment of inhibitory cAMP effectors, focusing on PKA type I (PKA-I), which interacts with the membrane-microfilament linker and AKAP, ezrin (27). Ezrin transiently accumulates at the IS, recruiting PKA to this location (25–27), but eventually moves toward the opposite side of the cell to the distal pole complex which includes inhibitory proteins such as CD43 and PKA (31, 47), allowing the TCR to signal at the IS.

**Figure 5:**
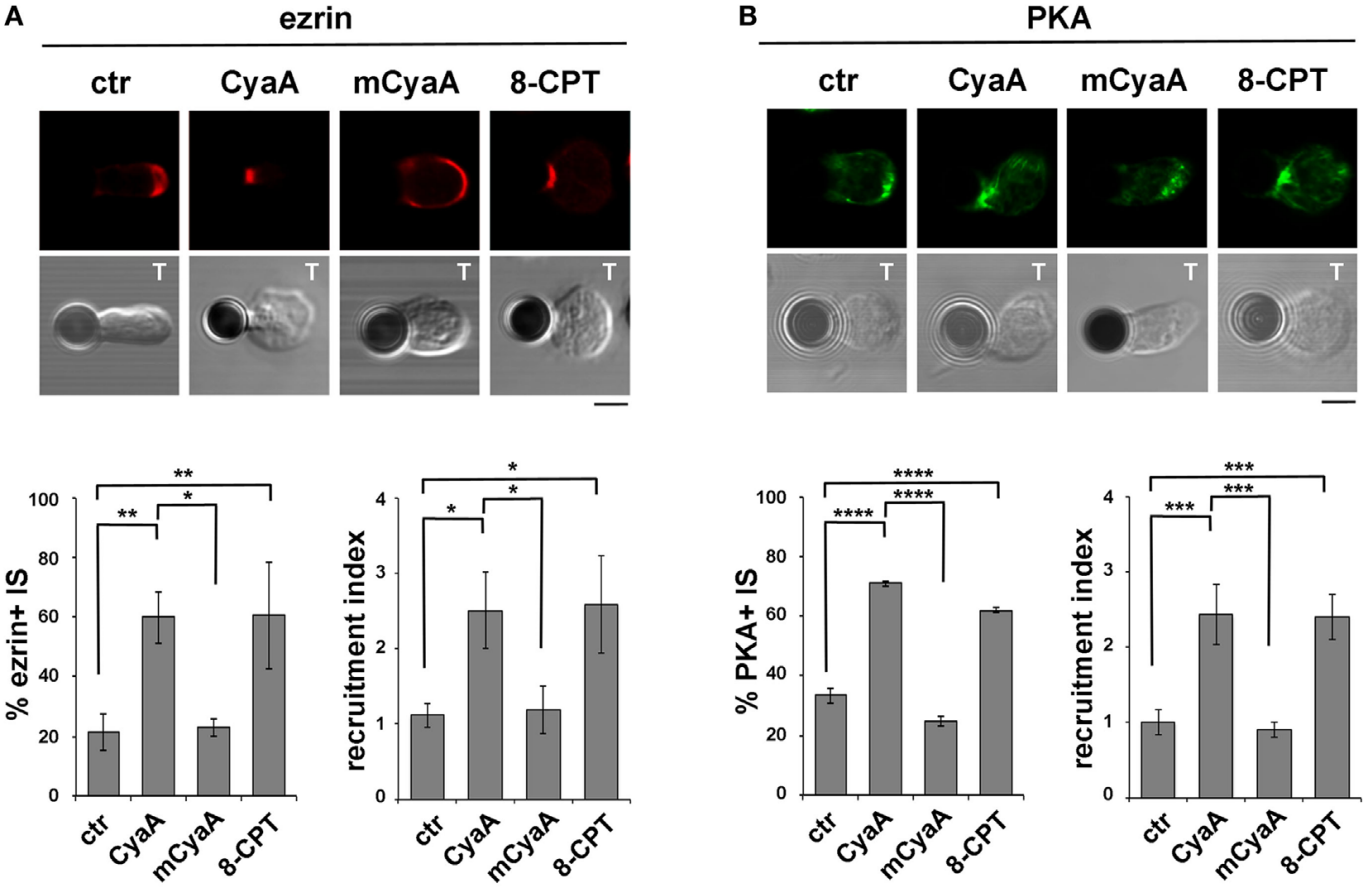
The ezrin-mediated recruitment of protein kinase A (PKA) to the immune synapse (IS) is enhanced in T cells intoxicated with CyaA. **(A,B)** Immunofluorescence analysis of ezrin **(A)** or PKA-I **(B)** in peripheral T cells incubated at 37°C for 15 min with beads coated with anti-CD3 and anti-CD28 mAbs and allowed to adhere on polylysine-coated wells for further 15 min. For intoxication T cells were incubated for 15 min on ice with 45 nM of either CyaA or mCyaA before washing and mixing with the beads. Ctrl cells were left untreated. Alternatively, 100 µM 8-CPT was added 2 min after T cells were mixed with the beads. Histograms show the percentage of conjugates harboring ezrin or PKA-I at the T cell-bead contact (mean ± SD, *n* = 3 donors; left) and the relative ezrin or PKA-I fluorescence at the T cell-bead contact compared with the remaining T cell area (expressed as relative recruitment index; right) (*n* = 45 cells, 15 cells/donor). Representative images are shown. Size bar, 5 µm. *P < 0.05; **P < 0.01; ***P < 0.005; ****P < 0.001. Error bars, SD (one-way ANOVA).

Due to the elevated expression of ezrin and PKA-I in Raji cells, which made it difficult to unambiguously track this molecule at the T cell side of the IS, T cells were conjugated with beads coated with anti-CD3 and anti-CD28 antibodies to form surrogate synapses. Confocal imaging of control conjugates showed a largely IS-distal localization of both ezrin and PKA-I, consistent with the strong synaptic accumulation of tyrosine phosphoproteins. Strikingly, ezrin (Figure 5A) and PKA-I (Figure 5B) were found associated with the IS in CyaA-intoxicated T cells, accounting for their defect in phosphotyrosine signaling. Similar results were obtained when T cells were treated with 8-CPT (Figures 5A,B). These effects were not reproduced by the AC-defective CyaA mutant (Figures 5A,B), indicating that they were mediated by cAMP. Hence phosphotyrosine signaling at the IS is dampened by the sustained presence of PKA-I, which can be activated by the local accumulation of cAMP elicited by CyaA. Interestingly, these data also provide evidence that the synaptic accumulation of ezrin is positively regulated by cAMP.

### The β-Arrestin-Mediated Recruitment of PDE4B at the IS Is Impaired in T Cells Intoxicated With CyaA

In addition to the ezrin-dependent relocalization of PKA at the distal pole away from the IS, T cells exploit an additional strategy to limit signaling at the IS. Namely, TCR coengagement with the costimulatory receptor CD28 has been reported to elicit the local degradation of cAMP by promoting the lipid raft recruitment and synaptic accumulation of the phosphodiesterase PDE4B through its constitutive interaction with β-arrestin (29).

**Figure 6:**
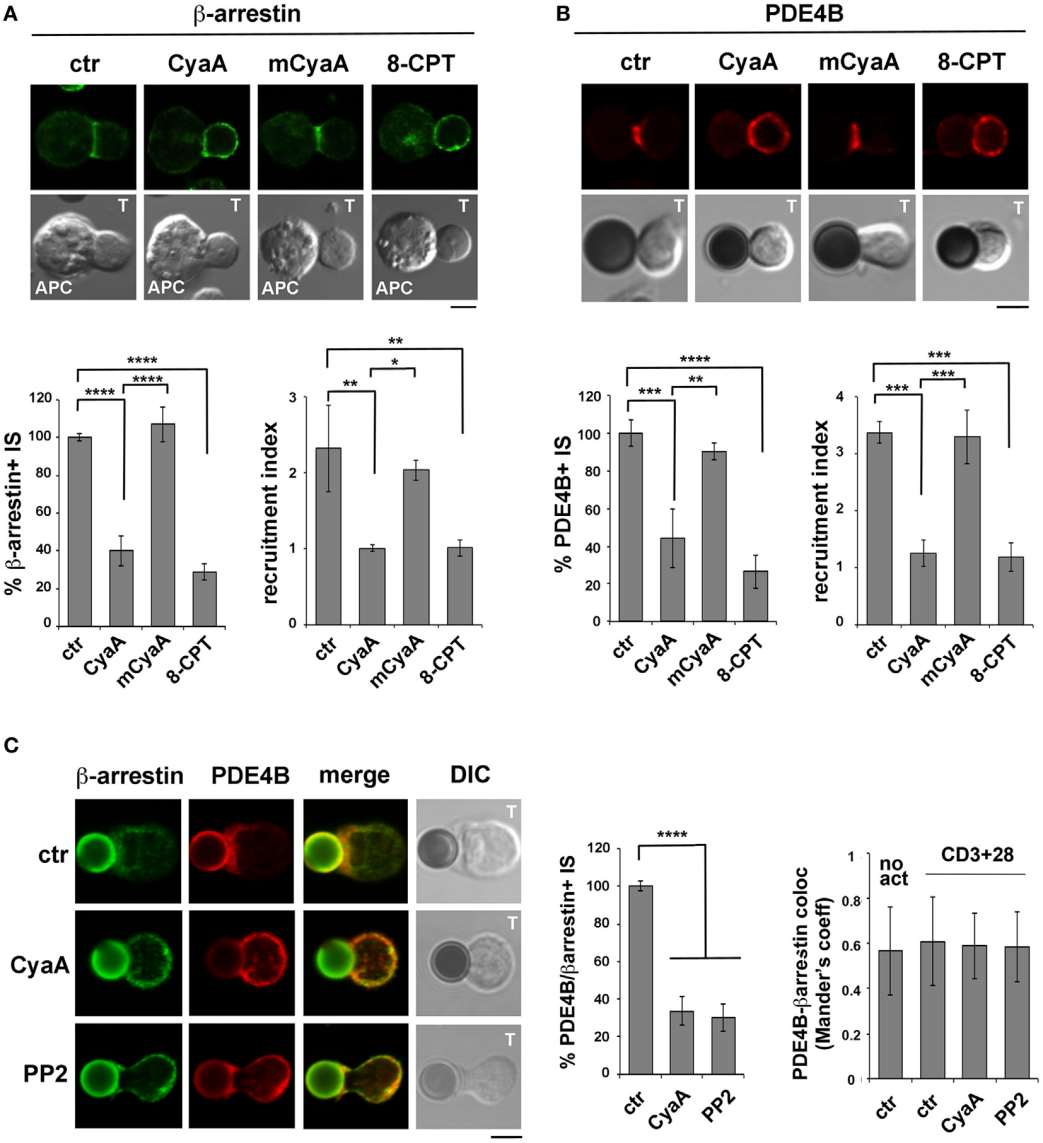
The β-arrestin-mediated recruitment of PDE4B at the immune synapse (IS) is impaired in T cells intoxicated with CyaA. Immunofluorescence analysis of β-arrestin **(A)** or PDE4B **(B)** in conjugates of peripheral T cells and SAg-pulsed antigen-presenting cell (APC) **(A)** or beads coated with anti-CD3 and anti-CD28 mAbs **(B)**, incubated at 37°C for 15 min and allowed to adhere on polylysine-coated wells for further 15 min. For intoxication T cells were incubated for 15 min on ice with 45 nM of either CyaA or mCyaA before washing and mixing with the APC. Ctrl cells were left untreated. Alternatively, 100 µM 8-CPT was added 2 min after T cells were mixed with APCs. Histograms show the percentage of conjugates harboring β-arrestin or PDE4B at the IS (mean ± SD, *n* = 3 donors; left) and the relative β-arrestin or PDE4B fluorescence at the T-cell:APC/bead contact site compared with the remaining T cell area (expressed as relative recruitment index; right) (*n* = 45 cells, 15 cells/donor). Due to the different efficiency of conjugate formation among donors, the percentages of synapses positive for each marker in treated vs untreated cells in each experiment have been normalized taking the value of untreated controls as 100%. The range of β-arrestin- and PDE4B-positive synapses in untreated controls from the three donors is ~30 and ~60%, respectively. **(C)** Colocalization analysis of β-arrestin and PDE4B in peripheral T cells incubated at 37°C for 15 min with beads coated with anti-CD3 and anti-CD28 mAbs (CD3 + 28) and allowed to adhere on polylysine-coated wells for further 15 min. For intoxication, T cells were incubated for 15 min on ice with 45 nM of CyaA before washing and mixing with the beads. Alternatively, 20 µM PP2 was added 2 min after T cells were mixed with the beads. Ctrl cells were treated with carrier. A sample of ctr cells was incubated at 37°C for 15 min without beads for the colocalization analysis of PDE4B and β-arrestin in resting cells (no act). Histograms show (left) the percentage of conjugates harboring both PDE4B and β-arrestin at the IS (mean ± SD, *n* = 3 donors) or (right) the colocalization of PDE4B and β-arrestin, quantified and expressed as Mander’s coefficient (mean ± SD, one-way ANOVA; *n* = 30 cells, 10 cells/donor, 3 donors). The percentages of synapses positive for each marker in treated vs untreated cells in each experiment have been normalized taking the value of untreated controls as 100%. The range of synapses positive for both PDE4B and β-arrestin in untreated controls from the three donors is ~50–60%. Representative images are shown. Size bar, 5 µm. *P < 0.05; **P < 0.01; ***P < 0.005; ****P < 0.001. Error bars, SD.

The impact of CyaA intoxication on the IS recruitment of β-arrestin and PDE4B was addressed by imaging conjugates of CyaA-intoxicated T cells with SAg-pulsed APCs (β-arrestin) or beads coated with anti-CD3 and anti-CD28 mAbs. At variance with control conjugates, which showed a strong accumulation of both β-arrestin (Figure 6A) and PDE4B (Figure 6B) at the IS, CyaA-treated cells showed a diffuse distribution of either molecule (Figures 6A,B). These effects were reproduced by 8-CPT but not by mCyaA (Figures 6A,B). Colocalization analyses showed moreover a strong synaptic colocalization of β-arrestin and PDE4B (Figure 6C). Consistent with their constitutive interaction (29), this colocalization was maintained after their dissipation away from the IS in CyaA-treated T cells (Figure 6C). These results indicate that cAMP activates a regulatory loop that displaces PDE4B and β-arrestin from the IS, thereby preventing cAMP degradation and thus suppressing TCR signaling.

To understand whether the abnormalities in distribution of the β-arrestin/PDE4B complex in CyaA-intoxicated T cells could be the consequence of premature termination of TCR signaling by cAMP/PKA through the Csk-mediated inhibition of the initiating kinase Lck (30), we imaged β-arrestin and PDE4B in T cells conjugated with beads coated with anti-CD3 and anti-CD28 mAbs and treated with the Src kinase inhibitor PP2 shortly after conjugate formation to allow for IS assembly (as assessed by staining with anti-PTyr mAb; Figure S5 in Supplementary Material). Under these conditions β-arrestin and PDE4B were found to dissipate together away from the IS, similar to CyaA-treated T cells (Figure 6C), supporting the notion that CyaA promotes the redistribution of the PDE4B-β-arrestin complex through its ability to inhibit Lck, thereby shutting off TCR signaling.

## Discussion

Using two bacterial AC toxins that catalyze the production of cAMP at different subcellular localizations as the result of their different routes for delivery into host cells, namely *B. pertussis* CyaA and *B. anthracis* ET (32, 33), here, we have addressed the outcome of cAMP compartmentalization on IS stability and function. We show that the IS is exquisitely susceptible to local cAMP domains and provide evidence that potent mechanisms for preventing cAMP diffusion away from the site of production are operational in T cells. We moreover characterize the mechanisms through which cAMP affects the architecture and function of the IS, identifying the pSMAC as a selective target of the suppressive effects of cAMP.

We had previously reported that CyaA exploits its ability to bind LFA-1 during IS assembly to concentrate at the pSMAC, wherefrom it is internalized into a subsynaptic compartment (14). This localization is ideally suited for the generation of a cAMP pool close to the IS membrane, where it can modulate signaling through PKA and other effectors. The ability of CyaA to promote the premature disengagement of LFA-1 from the IS (14) is likely the consequence of impaired signaling by the TCR, on which the conformational change required for talin binding depends (48, 49), resulting in talin dissipation and its uncoupling from the underlying actin cytoskeleton. Consistent with this notion, we found that phosphotyrosine signaling is severely impaired at the IS of CyaA-intoxicated T cells, notwithstanding the fact that the accumulation of the TCR at the IS is not affected by the toxin. Interestingly, a similar differential effect on LFA-1 and TCR clustering at the IS has been observed in T cells knocked down for L-plastin expression (42), suggesting that LFA-1 might dissipate faster from the IS compared to the TCR when signaling is turned off. Alternatively, the mechanism that controls the polarized transport of endosomal TCRs to the IS to replace internalized TCRs (50), that is expected to be triggered by microcluster-associated TCRs at the dSMAC, may be affected to a lesser extent due to compartmentalization of cAMP close to the site of production by CyaA, such that cAMP is prevented to reach the dSMAC. The fact that 8-CPT impairs sustained TCR clustering at the IS supports this notion. Indeed, as a membrane-permeable and diffusible non-hydrolyzable cAMP analog that is maintained at high concentrations throughout the cell, it has the potential to activate cAMP effectors at any subcellular localization, including the dSMAC. Together with premature depolymerization of synaptic F-actin, this may account for the impairment in F-actin accumulation at the IS observed in the presence of 8-CPT, but not of CyaA, since new actin filaments, on which the transport of TCR microclusters to the cSMAC depends, are polymerized at the dSMAC. Yet, we cannot rule out that in our experimental setting the synaptic concentration of 8-CPT may be higher than the cAMP concentration locally accumulated by CyaA, thereby accounting for the more profound effects of 8-CPT.

Although several steps of the TCR signaling cascade are potential targets of PKA, this kinase has been implicated in the initial step of TCR signaling, namely phosphorylation of the ITAMs within the CD3ζ subunit of the TCR complex by the kinase Lck (51). This is achieved through the PKA-I-mediated phosphorylation and resulting activation of the kinase Csk, which phosphorylates Lck on an inhibitory residue, thereby preventing TCR signaling (28). PKA-I undergoes a dynamic relocalization during IS assembly, being initially recruited by ezrin which is also responsible for its subsequent displacement away from the IS to the distant pole complex (27, 30). Our results showing that ezrin and PKA-I co-accumulate at the IS of CyaA-intoxicated T cells, at variance with control conjugates where they are found at the distal T cell pole, are consistent with the AKAP function of ezrin and indicate that this function allows for PKA to be recruited to the cAMP-enriched domain generated by CyaA at the IS, in a localization where TCR signaling can be modulated. Interestingly, they also provide evidence that the synaptic localization of ezrin can be stabilized by cAMP. Ezrin has been shown to be phosphorylated by PKA on a specific serine residue in gastric parietal cells (52). This event is critical for its participation in multimolecular complexes that provide spatial cues for H,K-ATPase trafficking and regulated HCl secretion (53). Phosphorylated ezrin restores moreover actin organization and PKA submembrane compartmentalization in cystic fibrosis airway cells, which corrects the chloride secretion defect observed in the presence of the trafficking-incompetent F508del mutant of the cystic fibrosis transmembrane regulator (54). This suggests that a feedback loop might be operational at the IS in the presence of cAMP, whereby PKA recruitment by ezrin, which associates with the pSMAC, would result in ezrin phosphorylation by PKA, reinforcing its interactions with synaptic molecular partners, such as the transmembrane adaptor PAG, ezrin binding protein 50 and Csk binding protein (55).

PDEs are central to the generation of cAMP domains, as elegantly demonstrated in cardiomyocytes and neurons, where they are recruited to the relevant subcellular localization by AKAPs in concert with β-arrestin (56). A similar mechanism has been identified in T cells, where TCR signaling promotes an elevation in cAMP which in the presence of CD28 costimulation is rapidly degraded by PDE4B, the latter forming a constitutive complex with β-arrestin at lipid rafts that is recruited to the IS (29). We found that CyaA not only catalyzes the local production of cAMP but also prevents its degradation by suppressing the β-arrestin-mediated recruitment of PDE4B at the IS, thereby allowing for robust and sustained PKA signaling. The defect in phosphotyrosine signaling observed in CyaA-intoxicated or 8-CPT-treated T cells is likely to account for the failure of β-arrestin-PDE4B to accumulate at the IS in these cells. The fact that a pharmacological Src inhibitor can recapitulate the effect of CyaA/8-CPT on the synaptic accumulation of β-arrestin-PDE4B supports this notion.

Surprisingly, ET did not reproduce the effects of CyaA at the IS, despite its ability to produce cAMP at comparable levels, consistent with the notion that cAMP remains compartmentalized close to the site of production (32, 33). This is set by the route of toxin delivery into the host cell, with CyaA remaining associated with or close to the plasma membrane, and ET trafficking to late endosome, where the low pH promotes the delivery of the catalytic subunit to the cytosolic side of the endosomal membrane, resulting in the generation of a perinuclear cAMP pool. The fact that the IS is selectively susceptible to CyaA indicates that T cells are able to maintain cAMP compartmentalized even when produced at supraphysiological concentrations, such as after intoxication with CyaA or ET, despite their scant cytoplasm. It is noteworthy that, notwithstanding their differential effects on the IS, both CyaA and ET suppress T cell activation and proliferation through their cAMP-elevating activity, as previously reported using antibody-mediated CD3 cross-linking as a means to trigger TCR signaling (9, 10, 12). Inhibition of both early and late TCR signaling was observed under these conditions (12). Here we show that when T cells are activated in the more physiological context of the IS, phosphotyrosine signaling at the IS is unaffected by ET, although key biological outcomes of TCR signaling, namely T cell activation and proliferation, are impaired following ET intoxication. Given the multiple steps in the TCR signaling cascade that can be modulated by cAMP this suggests that, while not affecting IS stability or local signaling, ET suppresses T cell activation by interfering with signaling downstream of the IS. In support of this notion, we found that the activation of MAP kinases, which is known to be inhibited by cAMP/PKA (57), is impaired in ET-treated T cells. These findings also imply that receptors that are coupled to endogenous ACs and known to modulate TCR signaling, such as prostaglandin receptors (58), may not interfere with signaling at the IS unless they are recruited to this location, but act downstream to affect TCR signaling and T cell activation, similar to ET. In this respect it could be speculated that Gi-coupled GPCRs such as CXCR4, which are recruited to the IS together with the TCR (59), might cooperate with PDE4B to sustain TCR signaling by limiting the local cAMP pool resulting from activation of TCR-coupled ACs.

## Ethics Statement

This study was carried out in accordance with the recommendations of Comitato Etico Regione Toscana—Area Vasta Sud Est on the use of human biological material (buffy coats from anonymous healthy donors) with written informed consent from all subjects. All subjects gave written informed consent in accordance with the Declaration of Helsinki. The protocol was approved by the Comitato Etico Regione Toscana—Area Vasta Sud Est.

## Author Contributions

VA, CU, AO, FF, and CB designed the experiments and analyzed the data; VA, CU, AO, and FF carried out the experiments; FT and DL provided key reagents and expertise. VA and CB wrote the paper.

## Conflict of Interest Statement

The authors declare that the submitted work was carried out in the absence of any personal, professional or financial relationships that could potentially be construed as a conflict of interest.
