# Supplementary material for: Compartmentalized Cyclic AMP Production by the Bordetella pertussis and Bacillus anthracis Adenylate Cyclase Toxins Differentially Affects the Immune Synapse in T Lymphocytes
Source: Front Immunol. 2018 May 1;9:919. doi: 10.3389/fimmu.2018.00919 (PMC5938339; doi:10.3389/fimmu.2018.00919)
Supplement: Supplementary file 1 [file image_1.PDF]

Supplementary figure legends

Figure S1. Control T cell:APC conjugates stained with the antibodies used in this report. Immunofluorescence analysis of conjugates of peripheral T cells and APC in the presence or absence of SAg. Conjugates were stained with the indicated antibodies. Representative images are shown. Size bar, 5 µm.

Figure S2. A. CyaA and ET catalyze cAMP production and inhibit TCR-dependent Erk activation in T cells. cAMP production in peripheral T cells intoxicated by incubation either for 30 min at 37°C with 45 nM CyaA or for 2 h at 37°C with ET (19 nM PA + 11 nM EF). The levels of cAMP, measured in T cell lysates, are expressed as fmoles/10^6 cells (mean±SD) (n=2). **B**. Immunoblot analysis of Erk1/2 phosphorylation in lysates of peripheral T cells activated for 15 min with SAg-pulsed Raji cells. The stripped filters were reprobed with anti-Erk2 antibody. The migration of molecular mass markers is indicated. The histogram shows the quantification of the P-Erk signal, normalized to Erk2 (mean±SD) (n=2).

Figure S3. F-Actin and TCR accumulate at the IS before the addition of 8-CPT. Immunofluorescence analysis of F-actin (**A**), CD3 (**B**) or PTyr (**C**) in conjugates of peripheral T cells and SAg-pulsed APC, incubated at 37°C for 2 min (second panel from left) or 15 min (last panel from left) and allowed to adhere on polylysine-coated wells for further 15 min. Ctrl cells (first panel from left) were added with carrier. Alternatively, 100 µM 8-CPT was added 2 min after T cells were mixed with APCs and allowed to adhere on polylysine-coated wells for further 13 min (third panel from left). Representative images obtained on a representative donor out of 3 are shown. The percentages of F-actin, CD3 or PTyr-positive conjugates were comparable at the 2 min and 15 min stimulation (mean ± SD F-actin: 72±3% 2 min *vs* 69±1.4% 15 min; CD3 74.3±3.2% 2 min *vs* 69±1.4% 15 min; PTyr 69.7±2.1% 2 min *vs* 71±1.4% 15 min, one-way ANOVA). Size bar, 5 µm.

Figure S4. Dose-response analysis of F-Actin and TCR accumulation at the IS in 8-CPT treated T cells. Immunofluorescence analysis of F-actin (**A**), CD3 (**B**) or PTyr (**C**) in conjugates of peripheral T cells and SAg-pulsed APC, incubated at 37°C for 15 min and allowed to adhere on polylysine-coated wells for further 15 min. Ctrl cells were added with carrier. Alternatively, 1 µM, 10 µM or 100 µM 8-CPT was added 2 min after T cells were mixed with APCs and allowed to adhere on polylysine-coated wells for further 13 min. Histograms show the percentage of conjugates harboring F-actin (**A**), CD3 (**B**) or PTyr (**C**) at the IS (mean ± SD, n=2 donors; left) and the relative F-actin (**A**), CD3 (**B**) or PTyr (**C**) fluorescence at the T-cell:APC contact site compared with the remaining T cell area (expressed as relative recruitment index; right) (n=30 cells, 15 cells/donor). Representative images are shown. Size bar, 5 µm. *$P < 0.05$; **$P < 0.01$; ***$P < 0.005$; ****$P < 0.001$ (one-way ANOVA).

Figure S5. Tyrosine phosphoproteins accumulate at the IS before the addition of PP2. Immunofluorescence analysis of PTyr in conjugates of peripheral T cells and SAg-pulsed APC, incubated at 37°C for 2 min (second panel from left) or 15 min (last panel from left) and allowed to adhere on polylysine-coated wells for further 15 min. Ctrl cells (first panel from left) were added with carrier. Alternatively, 20 µM PP2 was added 2 min after T cells were mixed with APCs and allowed to adhere on polylysine-coated wells for further 13 min (third panel from left). Representative images obtained on a representative donor out of 2 are shown. The percentages of PTyr-positive conjugates were comparable at the 2 min and 15 min stimulation (mean ± SD 76±1% 2 min *vs* 77.5±1% 15 min, one-way ANOVA). Size bar, 5 µm.

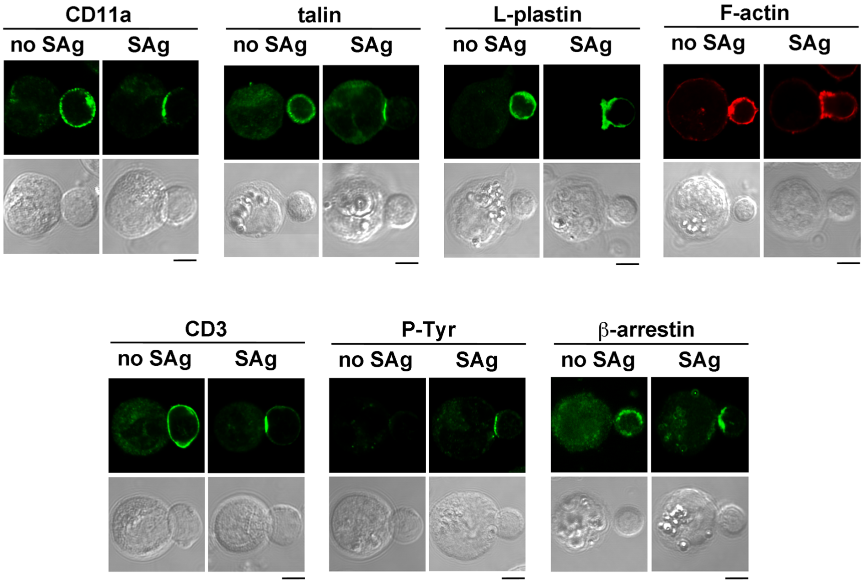
CD11a
no SAg
SAg
talin
no SAg
SAg
L-plastin
no SAg
SAg
F-actin
no SAg
SAg
CD3
no SAg
SAg
P-Tyr
no SAg
SAg
β-arrestin
no SAg
SAg

FIGURE S1

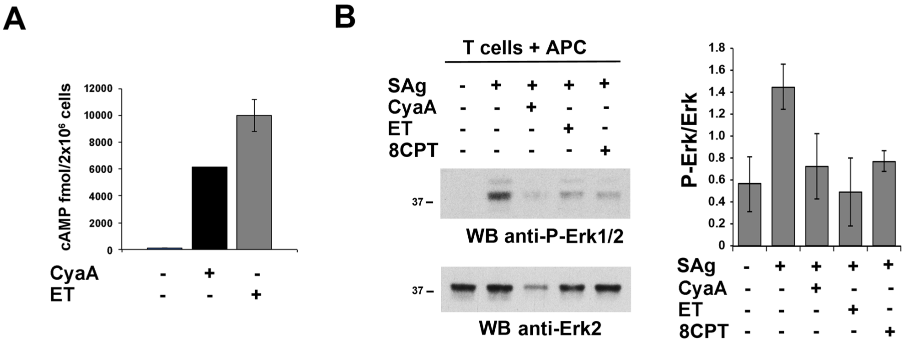
A
B
cAMP fmol/2x10⁶ cells
12000
10000
8000
6000
4000
2000
0
CyaA - + -
ET - - +
T cells + APC
SAg - + + + +
CyaA - - + - -
ET - - - + -
8CPT - - - - +
37
WB anti-P-Erk1/2
37
WB anti-Erk2
P-Erk/Erk
1.8
1.6
1.4
1.2
1.0
0.8
0.6
0.4
0.2
0
SAg - + + + +
CyaA - - + - -
ET - - - + -
8CPT - - - - +

FIGURE S2

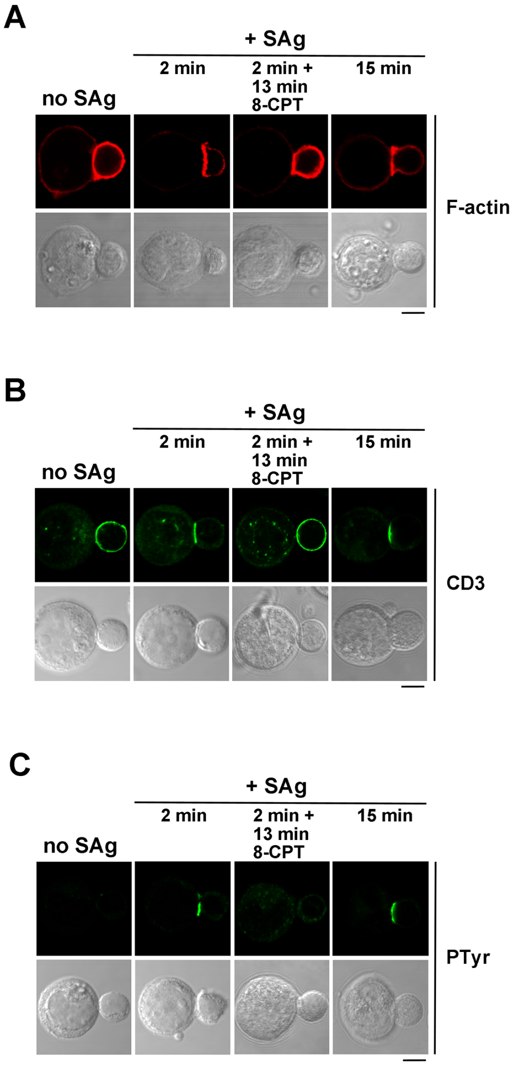
A
+ SAg
2 min
2 min + 13 min 8-CPT
15 min
no SAg
F-actin
B
+ SAg
2 min
2 min + 13 min 8-CPT
15 min
no SAg
CD3
C
+ SAg
2 min
2 min + 13 min 8-CPT
15 min
no SAg
PTyr

FIGURE S3

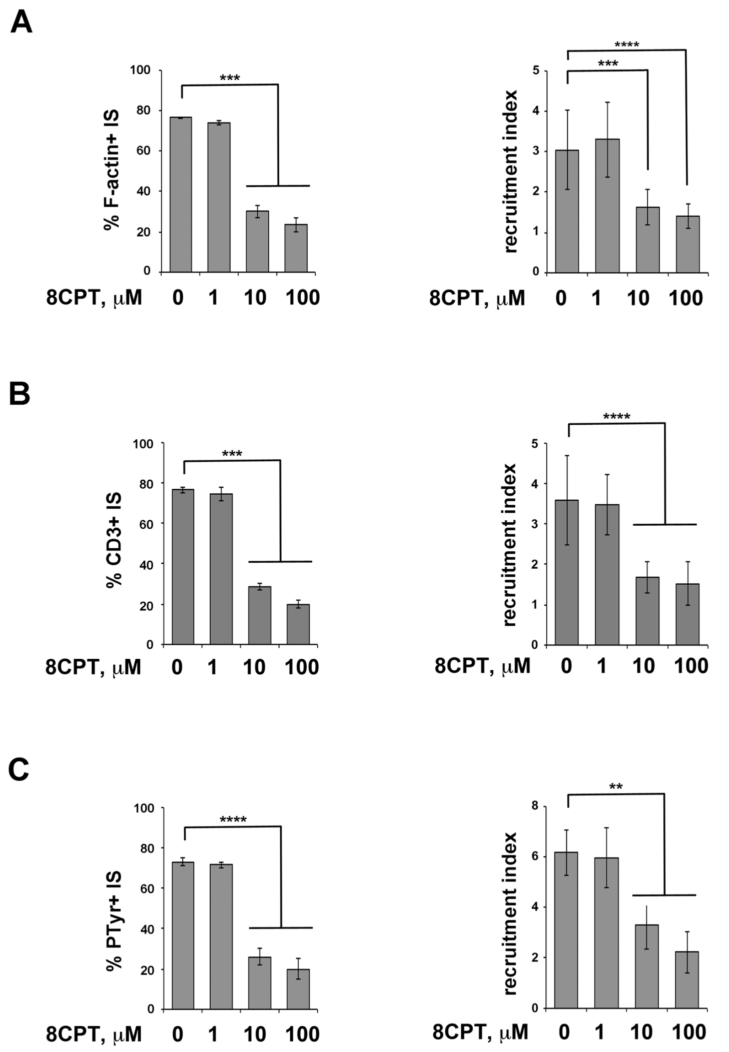
A
B
C
% F-actin+ IS
% CD3+ IS
% PTyr+ IS
recruitment index
8CPT, μM
0
1
10
100
***
****
**

FIGURE S4

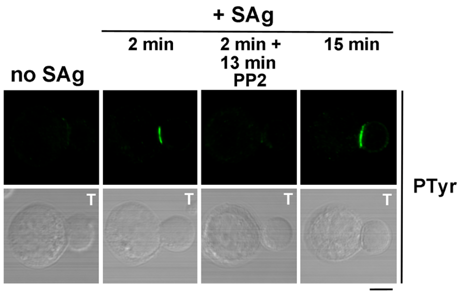

FIGURE S5
